# Racial disparities in maternal blood transfusion in the United States by mode of delivery

**DOI:** 10.1371/journal.pone.0312110

**Published:** 2024-10-21

**Authors:** Parnian Hossein-Pour, Maya Rajasingham, Michelle P. Zeller, Giulia M. Muraca

**Affiliations:** 1 Department of Obstetrics and Gynecology, Faculty of Health Sciences, McMaster University, Hamilton, ON, Canada; 2 Department of Health Research Methods, Evidence, and Impact, Faculty of Health Sciences, McMaster University, Hamilton, ON, Canada; 3 Canadian Blood Services, Ottawa, Ontario, Canada; 4 Michael G. DeGroote Centre for Transfusion Research, McMaster University, Hamilton, Ontario, Canada; 5 Department of Medicine, McMaster University, Hamilton, Ontario, Canada; 6 Clinical Epidemiology Unit, Department of Medicine, Solna, Karolinska Institutet, Stockholm, Sweden; Delta State University, NIGERIA

## Abstract

**Background:**

Despite well-documented racial disparities in maternal health in the United States, gaps remain in characterizing the distribution of these disparities in maternal blood transfusion.

**Objective:**

To assess racial disparities in maternal blood transfusion using detailed, self-identified racial groupings in the United States overall and stratified by mode of delivery.

**Study design:**

We performed a population-based, retrospective cohort study of full term, live births (2016–2021) using the National Vital Statistics System’s Natality Files. Regression models were constructed to estimate adjusted odds ratios (aORs) and 95% confidence intervals (CIs) of maternal blood transfusion by self-identified maternal race in the total population, and among subgroups stratified by mode of delivery. Models were adjusted for maternal and obstetric practice factors.

**Results:**

The study included 17,905,699 deliveries; maternal blood transfusion occurred in 3.4 per 1,000 deliveries. Compared with individuals who identified as White (3.3 per 1,000 transfusion rate), higher odds of transfusion were found among those who identified as American Indian and Alaska Native (AIAN; aOR 2.36, 95% CI 2.23–2.49), Black (aOR 1.15, 95% CI 1.12–1.17), Filipino (aOR 1.33, 95% CI 1.22–1.44), Korean (aOR 1.25, 95% CI 1.10–1.42), and Pacific Islander (aOR 1.63, 95% CI 1.45–1.83). The frequency of transfusion and racial disparities in transfusion varied substantially by mode of delivery. Lower rates of transfusion in Black vs White patients were observed in the spontaneous vaginal delivery (2.2 vs 2.3 per 1000; aOR 0.95, 95% CI 0.92–0.99), forceps (6.8 vs 8.9 per 1000; aOR 0.77, 95% CI 0.60–0.99), vacuum (4.2 vs 5.0 per 1000; aOR 0.85, 95% CI 0.74–0.97, and cesarean delivery with trial of labour (8.8 vs 8.9 per 1000; aOR 0.95, 95% CI 0.91–1.00) groups, while a higher rate was shown among cesarean deliveries without trial of labour (6.8 vs 4.3 per 1000; aOR 1.45, 95% CI 1.40–1.51).

**Conclusion:**

Racial disparities in maternal blood transfusion persist after adjustment for several confounders, particularly for AIAN and Pacific Islander individuals, and vary by mode of delivery.

## Introduction

Obstetric hemorrhage is a leading cause of maternal mortality worldwide and its treatment with blood transfusion support is imperative for maternal survival [1]. Etiology of obstetric hemorrhage include uterine atony, lacerations of the genital tract, uterine rupture, uterine inversion, retained product, chorioamnionitis, and subinvolution of the uterus [2–4]. Obstetric hemorrhage affects approximately 3% of births [5, 6]; though obstetric hemorrhages requiring a blood transfusion are less frequent, with an incidence rate of approximately 0.3% in the United States (US) [7]. Notably, maternal blood transfusion can also be indicated for other causes of antepartum, peripartum and postpartum anemia [8].

While maternal blood transfusion may be a lifesaving procedure, there remains risk to both mother and fetus/infant from potential adverse transfusion reactions [9]. As such, it is critical that transfusions are provided when clinically indicated. Blood transfusion is a limited resource; it is incumbent on providers to use blood components equitably, judiciously and in accordance with best-practices and available guidelines.

One key risk factor for a maternal blood transfusion is the mode of delivery [8, 9]. Operative vaginal deliveries and cesarean deliveries have been shown to increase the risk of transfusion compared with spontaneous vaginal deliveries (SVDs), with the highest rates among forceps deliveries and cesarean deliveries following trial of labour (TOL) [10, 11]. Rates of obstetric hemorrhage have also been shown to vary by maternal race [10–12]. In general, racial/ethnic minorities experience higher rates of maternal transfusion compared to White individuals [10, 12]. However, the literature examining racial disparities in obstetric hemorrhage is frequently constrained by aggregated race/ethnicity categories. Given that these categories assume homogeneity, important disparities and potential determinants of inequities regarding maternal transfusion may be masked [12–14]. It is important to note, however, that though self-identified race is the variable of interest in this work, we are not measuring the impacts of a biologic phenomenon [15]. Rather, we acknowledge that race is instead a social and cultural context whose value in this analysis is to serve as a proxy for structural and systematic racism [16, 17].

The aim of this study was to assess racial disparities in maternal blood transfusion in the US overall and stratified by mode of delivery using granular indicators of self-reported maternal race.

## Methods

### Study population and data source

We conducted a population-based, retrospective cohort study including all US live births between 37 and 42 weeks of gestation. Data was obtained from the publicly-accessible National Vital Statistics System’s (NVSS) Natality File which collects information on approximately 99% of all registered births in the United States [18]. Data were abstracted from birth certificates that had been completed by the birth attendant following a standardized guide and include details on maternal and infant characteristics including information on demographics, comorbidities, and obstetric practice factors [18]. Race information was self-reported and collected through a Mother’s Worksheet at the time of birth [18]. In instances where the mother’s race has not been reported, the race of the father was assigned to the mother in the birth certificate, if that information was available [18]. If this was also unavailable, a past certificate with a known maternal race was sought and used [18].

This study was restricted to the years 2016–2021 as prior to 2016 birth certificate race reporting was not consistent across all states. To account for differences in variable coding over the study years, all variables were recoded to the 2021 equivalent.

### Exposure and outcome variables: Maternal race and maternal transfusion

The exposure, self-reported maternal race, was categorized by the NVSS Natality File into American Indian or Alaska Native (AIAN), Black, Chinese, Filipino, Guamanian, Asian Indian, Japanese, Korean, more than one race, Native Hawaiian, other Asian, other Pacific Islander, Samoan, Vietnamese, and White. In this analysis, Guamanian, Native Hawaiian, Samoan, and other Pacific Islander were grouped to form a Pacific Islander group due to their respective small populations. Thus, 12 race categories were considered in this analysis.

The outcome measure, maternal blood transfusion, was a binary variable indicating whether the participant received any form of blood transfusion (whole blood or packed red blood cells) during labour and delivery [18]. The type of transfusion was not available in the data source and thus not included in this analysis. Data were recorded in the NVSS Natality File based on delivery record, physician delivery notes, or intake and output form [18]. Analyses were stratified by mode of delivery; the delivery modes were SVD, forceps delivery, vacuum delivery, cesarean delivery with TOL, and cesarean delivery without TOL.

### Statistical analysis

Demographic and clinical characteristics were compared by self-reported maternal race. Unadjusted rates of maternal blood transfusion were calculated by race in the overall population and by mode of delivery. Multivariable logistic regression was then used to examine the association between maternal race and receipt of blood transfusion in the overall population as well as stratified by mode of delivery while adjusting for several covariates. The covariates included in the regression models were identified based on prior literature and consisted of demographic and clinical characteristics associated with maternal blood transfusion and race. As such, the following covariates were included in all regression models: maternal age (< 15, 15–19, 20–24, 35–44, 45+ vs. 25–34 years), maternal education (8th grade or less, some high school, high school diploma, some college, Associate’s degree, Bachelor’s degree, Master’s degree vs. doctorate or professional degree), receipt of Women, Infants, and Children (WIC) food program, maternal nativity (born outside the United States vs. born inside the United States), parity (one prior delivery, two to three deliveries, more than four prior deliveries vs. zero prior deliveries), maternal pre-pregnancy BMI (< 18.5 kg/m^2^, 25.0–29.9 kg/m^2^, ≥ 30 kg/m^2^, vs. 18.5–24.9 kg/m^2^), multiple gestation, previous cesarean delivery, gestational hypertension, preeclampsia, eclampsia, pregestational hypertension, gestational diabetes, chorioamnionitis, labour induction, labour augmentation, and birth weight (≥ 4,000g vs. < 4,000g). A complete-case analysis was conducted. Regression results were reported as adjusted odds ratios (aORs) and 95% confidence intervals (CIs).

### Sensitivity analyses

A limitation of the NVSS Natality File is the lack of an identifier for the mother. Each observation denotes a birth and a repeated mother cannot be identified across observations and thus cannot be controlled for using generalized estimating equations. To account for the possibility that cases of maternal transfusion were clustered among deliveries by the same mother, we conducted a sensitivity analysis using only nulliparous patients. Adjusted logistic regression models identical to those described above were produced using this subset of the study population and results were reported as aORs with 95% CIs.

Additionally, birth certificate data have been shown to have low sensitivity for maternal morbidities [19]. To account for this, we performed a probabilistic sensitivity analysis to correct for potential outcome misclassification. We assumed a uniform distribution of sensitivity of blood transfusion between 10 and 50%, specificity between 99.8% and 100%, and nondifferential misclassification of blood transfusion between different races. Corrected odds ratios and 95% confidence intervals were generated using 500,000 iterations.

All analyses were performed on publicly accessible deidentified data and did not require ethics approval. They were conducted using R statistical software and the EpiDisplay and episensr packages [20–22]. This study followed the Strengthening the Reporting of Observational Studies in Epidemiology (STROBE) reporting guidelines.

## Results

**Fig 1:**
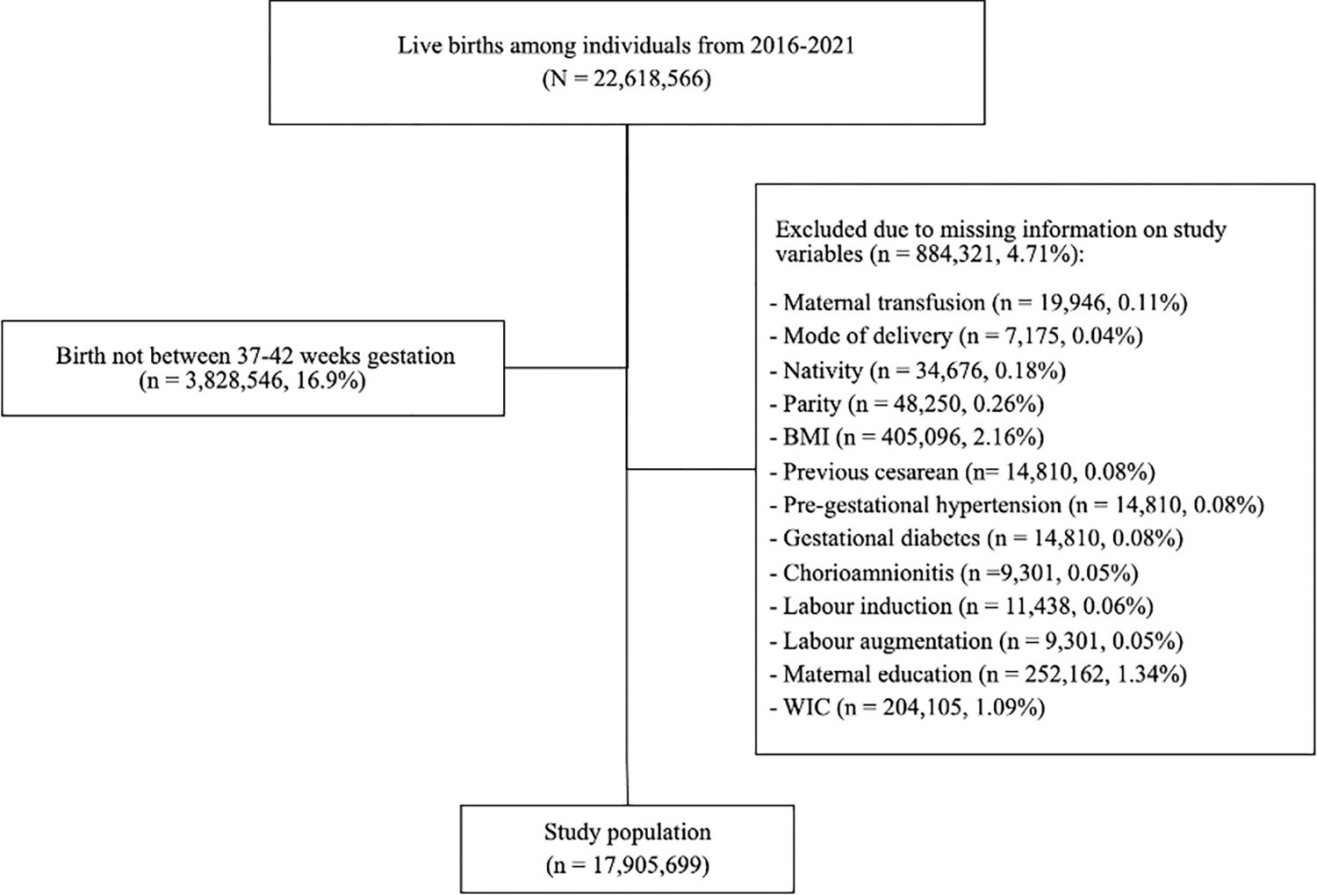
Derivation of study cohort.

There were 22,618,566 live births in the US between 2016 and 2021. After exclusions for births outside of 37–42 weeks’ gestation and births with missing covariate data, the study population included 17,905,699 live births from 2016–2021 (Fig 1). Demographics and clinical characteristics are outlined in detail in Table 1. Of the total population, 74.5% of patients were White (n = 13,343,790), 14.9% were Black (n = 2,669,131), 1.5% were Chinese (n = 269,920), 2.0% were Indian (n = 362,616), 2.7% identified with more than one race (n = 474,647), and 1.3% identified as other Asian (n = 229,487); the remaining race groups (AIAN, Filipino, Japanese, Korean, Pacific Islander, and Vietnamese) each represented less than 1% of the total population. Approximately 66.9% of all deliveries were SVDs, 0.5% were forceps deliveries, 2.7% were vacuum deliveries, 8.5% were cesarean deliveries with TOL, and 21.5% were cesarean deliveries without TOL.

**Table 1:** Demographic and clinical characteristics of individuals with a term birth in the United States, 2016–2021, (N = 17,905,699)^a^.

	Self-Identified Maternal Race											
	White	AIAN	Black	Chinese	Filipino	Indian	Japanese	Korean	>1 race	Other Asian	Pacific Islander	Vietnamese
N	13,343,790	162,489	2,669,131	269,920	145,049	362,616	30,756	69,052	474,647	229,487	50,944	97,818
Patient Demographics												
Maternal Age												
Under 15	4,406 (<0.1%)	162 (0.1%)	2,416 (0.1%)	<10 (<0.1%)	20 (<0.1%)	14 (<0.1%)	<10 (<0.1%)	<10 (<0.1%)	392 (0.1%)	30 (<0.1%)	23 (0.1%)	<10 (<0.1%)
15–19	560,297 (4.2%)	14,648 (9.0%)	181,670 (6.8%)	802 (0.3%)	1,525 (1.1%)	1,401 (0.4%)	106 (0.3%)	266 (0.4%)	36,238 (7.6%)	4,233 (1.8%)	2,880 (5.7%)	735 (0.8%)
20–24	2,444,973 (18.3%)	45,129 (27.8%)	672,921 (25.2%)	9,470 (3.5%)	11,546 (8.0%)	18,090 (5.0%)	876 (2.9%)	1,578 (2.3%)	123,861 (26.1%)	28,671 (12.5%)	12,829 (25.2%)	6,549 (6.7%)
25–34	7,931,753 (59.4%)	83,276 (51.3%)	1,411,077 (52.9%)	172,827 (64.0%)	83,196 (57.4%)	259,938 (71.7%)	14,827 (48.2%)	37,176 (53.8%)	246,574 (52.0%)	142,492 (62.1%)	27,557 (54.1%)	60,627 (62.0%)
35–44	2,377,421 (17.8%)	19,138 (11.8%)	395,373 (14.8%)	85,316 (31.6%)	48,160 (33.2%)	82,493 (22.8%)	14,574 (47.4%)	29,709 (43.0%)	66,827 (14.1%)	53,322 (23.2%)	7,573 (14.9%)	29,437 (30.1%)
45 and over	24,940 (0.2%)	136 (0.1%)	5,674 (0.2%)	1,497 (0.6%)	602 (0.4%)	680 (0.2%)	372 (1.2%)	322 (0.5%)	755 (0.2%)	739 (0.3%)	82 (0.2%)	465 (0.5%)
Maternal Education												
8th grade or less	436,251 (3.3%)	4,517 (2.8%)	54,661 (2.1%)	4,853 (1.8%)	953 (0.7%)	5,171 (1.4%)	140 (0.5%)	443 (0.6%)	4,651 (1.0%)	15,386 (6.7%)	2,794 (5.5%)	2,236 (2.3%)
Some high school	1,133,086 (8.5%)	30,288 (18.6%)	300,151 (11.3%)	10,393 (3.9%)	3,750 (2.6%)	10,518 (2.9%)	567 (1.8%)	676 (1.0%)	49,524 (10.4%)	18,893 (8.2%)	8,938 (17.5%)	5,943 (6.1%)
High school diploma	3,219,933 (24.1%)	58,790 (36.2%)	934,830 (35.0%)	25,603 (9.5%)	17,447 (12.0%)	27,646 (7.6%)	2,637 (8.6%)	3,374 (4.9%)	129,370 (27.3%)	49,595 (21.6%)	18,624 (36.6%)	21,390 (21.9%)
Some college	2,559,839 (19.2%)	40,328 (24.8%)	674,165 (25.3%)	21,213 (7.9%)	27,844 (19.2%)	17,911 (4.9%)	3,053 (9.9%)	5,982 (8.7%)	127,802 (26.9%)	32,356 (14.1%)	11,633 (22.8%)	15,439 (15.8%)
Associate degree	1,174,766 (8.8%)	12,202 (7.5%)	211,004 (7.9%)	14,354 (5.3%)	15,843 (10.9%)	12,129 (3.3%)	3,340 (10.9%)	3,741 (5.4%)	40,153 (8.5%)	16,715 (7.3%)	3,655 (7.2%)	8,774 (9.0%)
Bachelor’s degree	3,056,774 (22.9%)	11,790 (7.3%)	323,760 (12.1%)	90,761 (33.6%)	62,910 (43.4%)	129,844 (35.8%)	14,349 (46.7%)	31,112 (45.1%)	79,390 (16.7%)	58,982 (25.7%)	4,073 (8.0%)	28,251 (28.9%)
Master’s degree	1,375,269 (10.3%)	3,631 (2.2%)	139,528 (5.2%)	70,794 (26.2%)	11,506 (7.9%)	126,654 (34.9%)	4,756 (15.5%)	14,832 (21.5%)	32,763 (6.9%)	26,468 (11.5%)	989 (1.9%)	8,436 (8.6%)
Doctorate or professional degree	387,872 (2.9%)	943 (0.6%)	31,032 (1.2%)	31,949 (11.8%)	4,796 (3.3%)	32,743 (9.0%)	1,914 (6.2%)	8,892 (12.9%)	10,994 (2.3%)	11,092 (4.8%)	238 (0.5%)	7,349 (7.5%)
Mother born in United States	10,944,119 (82.0%)	149,944 (92.3%)	2,138,894 (80.1%)	42,986 (15.9%)	44,739 (30.8%)	40,311 (11.1%)	9,096 (29.6%)	18,611 (27.0%)	427,376 (9<0.1%)	70,079 (30.5%)	20,090 (39.4%)	23,684 (24.2%)
Mother Received WIC	4,150,941 (31.1%)	86,272 (53.1%)	1,431,830 (53.6%)	47,642 (17.7%)	29,448 (20.3%)	49,258 (13.6%)	2,891 (9.4%)	6,864 (9.9%)	185,150 (39.0%)	86,860 (37.9%)	22,033 (43.3%)	22,840 (23.4%)
Clinical Characteristics												
Parity												
0	5,108,392 (38.3%)	52,106 (32.1%)	970,397 (36.4%)	134,468 (49.8%)	60,780 (41.9%)	172,000 (47.4%)	14,316 (46.6%)	34,019 (49.3%)	199,705 (42.1%)	87,840 (38.3%)	15,828 (31.1%)	43,541 (44.5%)
1	4,383,008 (32.9%)	44,350 (27.3%)	778,696 (29.2%)	103,981 (38.5%)	50,753 (35.0%)	150,546 (41.5%)	11,514 (37.4%)	25,123 (36.4%)	143,655 (30.3%)	75,118 (32.7%)	13,104 (25.7%)	35,569 (36.4%)
2–3	3,234,467 (24.2%)	48,481 (29.8%)	727,105 (27.2%)	29,840 (11.1%)	30,394 (21.0%)	37,016 (10.2%)	4,576 (14.9%)	9,204 (13.3%)	108,316 (22.8%)	54,954 (24.0%)	15,590 (30.6%)	17,386 (17.8%)
4+	617,923 (4.6%)	17,552 (10.8%)	192,933 (7.2%)	1,631 (0.6%)	3,122 (2.2%)	3,054 (0.8%)	350 (1.1%)	706 (1.0%)	22,971 (4.8%)	11,575 (5.0%)	6,422 (12.6%)	1,322 (1.4%)
Maternal BMI												
Below 18.5	368,666 (2.8%)	3,370 (2.1%)	76,310 (2.9%)	31,057 (11.5%)	5,920 (4.1%)	14,580 (4.0%)	2,855 (9.3%)	4,971 (7.2%)	15,301 (3.2%)	10,523 (4.6%)	856 (1.7%)	9,883 (10.1%)
18.5–24.9	5,742,017 (43.0%)	50,330 (31.0%)	856,421 (32.1%)	191,962 (71.1%)	77,906 (53.7%)	194,623 (53.7%)	21,371 (69.5%)	47,550 (68.9%)	188,640 (39.7%)	118,808 (51.8%)	13,407 (26.3%)	67,959 (69.5%)
25–29.9	3,604,803 (27.0%)	45,363 (27.9%)	732,179 (27.4%)	36,752 (13.6%)	39,890 (27.5%)	109,737 (30.3%)	4,535 (14.8%)	12,093 (17.5%)	124,649 (26.3%)	64,143 (28.0%)	13,978 (27.4%)	14,662 (15.0%)
30 or greater	3,628,304 (27.2%)	63,426 (39.0%)	1,004,221 (37.6%)	10,149 (3.8%)	21,333 (14.7%)	43,676 (12.0%)	1,995 (6.5%)	4,438 (6.4%)	146,057 (30.8%)	36,013 (15.7%)	22,703 (44.6%)	5,314 (5.4%)
Multiple gestation	207,179 (1.6%)	2,161 (1.3%)	49,417 (1.9%)	4,489 (1.7%)	1,731 (1.2%)	5,176 (1.4%)	564 (1.8%)	1,017 (1.5%)	7,726 (1.6%)	2,487 (1.1%)	678 (1.3%)	1,099 (1.1%)
Pre-gestational hypertension	219,365 (1.6%)	3,706 (2.3%)	91,886 (3.4%)	1,418 (0.5%)	3,135 (2.2%)	3,054 (0.8%)	272 (0.9%)	696 (1.0%)	9,728 (2.1%)	2,578 (1.1%)	797 (1.6%)	617 (0.6%)
Gestational hypertension, preeclampsia, eclampsia	899,395 (6.7%)	13,154 (8.1%)	208,100 (7.8%)	6,873 (2.6%)	10,671 (7.4%)	13,981 (3.9%)	1,196 (3.9%)	2,624 (3.8%)	34,042 (7.2%)	9,979 (4.4%)	3,605 (7.1%)	3,046 (3.1%)
Previous cesarean	1,994,471 (15.0%)	24,695 (15.2%)	459,302 (17.2%)	34,700 (12.9%)	22,870 (15.8%)	65,719 (18.1%)	3,172 (10.3%)	8,628 (12.5%)	65,336 (13.8%)	33,080 (14.4%)	8,610 (16.9%)	13,737 (14.0%)
Gestational diabetes	861,990 (6.5%)	16,244 (1<0.1%)	149,810 (5.6%)	30,965 (11.5%)	18,715 (12.9%)	53,470 (14.8%)	2,342 (7.6%)	6,592 (9.6%)	30,717 (6.5%)	29,171 (12.7%)	4,657 (9.1%)	12,818 (13.1%)
Chorioamnionitis	190,009 (1.4%)	2,309 (1.4%)	42,069 (1.6%)	9,034 (3.4%)	5,913 (4.1%)	11,485 (3.2%)	800 (2.6%)	2,201 (3.2%)	9,094 (1.9%)	6,605 (2.9%)	969 (1.9%)	3,349 (3.4%)
Obstetric Care												
Labour induction	4,075,887 (30.6%)	49,202 (30.3%)	754,563 (28.3%)	57,929 (21.5%)	31,929 (22.0%)	97,009 (26.8%)	7,200 (23.4%)	17,607 (25.5%)	141,589 (29.8%)	56,001 (24.4%)	11,636 (22.8%)	19,459 (19.9%)
Labour augmentation	2,957,687 (22.2%)	38,816 (23.9%)	569,066 (21.3%)	71,114 (26.4%)	33,222 (22.9%)	91,383 (25.2%)	8,053 (26.2%)	17,692 (25.6%)	113,986 (24.0%)	58,922 (25.7%)	12,906 (25.3%)	23,463 (24.0%)
Mode of delivery												
Spontaneous vaginal	9,030,320 (67.7%)	114,894 (70.7%)	1,704,281 (63.9%)	181,281 (67.2%)	89,924 (62.0%)	205,006 (56.5%)	21,898 (71.2%)	45,376 (65.7%)	326,341 (68.8%)	155,468 (67.8%)	34,678 (68.1%)	63,582 (65.0%)
Forceps	72,165 (0.5%)	605 (0.4%)	10,910 (0.4%)	1,742 (0.7%)	939 (0.7%)	3,181 (0.9%)	335 (1.1%)	564 (0.8%)	2,493 (0.5%)	1,577 (0.7%)	291 (0.6%)	608 (0.6%)
Vacuum	346,648 (2.6%)	3,347 (2.1%)	60,793 (2.3%)	13,385 (5.0%)	5,593 (3.9%)	18,560 (5.1%)	1,178 (3.8%)	3,208 (4.7%)	12,509 (2.6%)	8,468 (3.7%)	1,142 (2.2%)	4,715 (4.8%)
Caesarean with TOL	1,074,373 (8.1%)	275,299 (10.3%)	16,759 (6.2%)	13,762 (9.5%)	42,311 (11.7%)	1,883 (6.1%)	5,590 (8.1%)	41,739 (8.8%)	20,455 (8.9%)	4,222 (8.3%)	7,653 (7.8%)	275,299 (10.3%)
Caesarean without TOL	2,820,284 (21.1%)	30,862 (19.0%)	617,848 (23.2%)	56,753 (21.0%)	34,831 (24.0%)	93,558 (25.8%)	5,462 (17.8%)	14,314 (20.7%)	91,565 (19.3%)	43,519 (19.0%)	10,611 (20.8%)	21,260 (21.7%)
Infant Characteristics												
Birth weight greater than 4000 g	1,226,678 (9.2%)	17,049 (10.5%)	130,592 (4.9%)	12,338 (4.6%)	7,296 (5.0%)	13,076 (3.6%)	1,092 (3.6%)	4,008 (5.8%)	36,830 (7.8%)	11,197 (4.9%)	5,054 (9.9%)	3,361 (3.4%)

^a^ Values are displayed as column totals and percentages; n (%).

AIAN, American Indian or Alaskan Native; >1 race, more than one race; BMI, body mass index; WIC, Women, Infants, and Children; TOL, trial of labour.

Individuals from race groups originating in Asia (Chinese, Filipino, Indian, Japanese, Korean, Vietnamese, and other Asian) had the highest average maternal age while White, AIAN, Black, and Pacific Islander individuals tended to be younger. Chinese, Indian, Japanese, and Korean groups had the highest rates of nulliparity. Mode of delivery differed by race, where Indian individuals had the lowest rate of SVD, Japanese individuals had the highest rate of forceps delivery, Chinese and Indian individuals had the highest rates of vacuum delivery, AIAN, Chinese, and Vietnamese individuals had the highest rates of cesarean delivery with TOL, and Indian individuals had the highest rate of cesarean without TOL. The highest prevalence of obesity (BMI ≥ 30 kg/m^2^) was seen in Pacific Islander patients, followed by AIAN and Black patients. Macrosomia also varied by maternal race, where AIAN, White, and Pacific Islander individuals had the highest rates (Table 1).

### Crude rate of maternal blood transfusion by maternal race and mode of delivery

**Fig 2:**
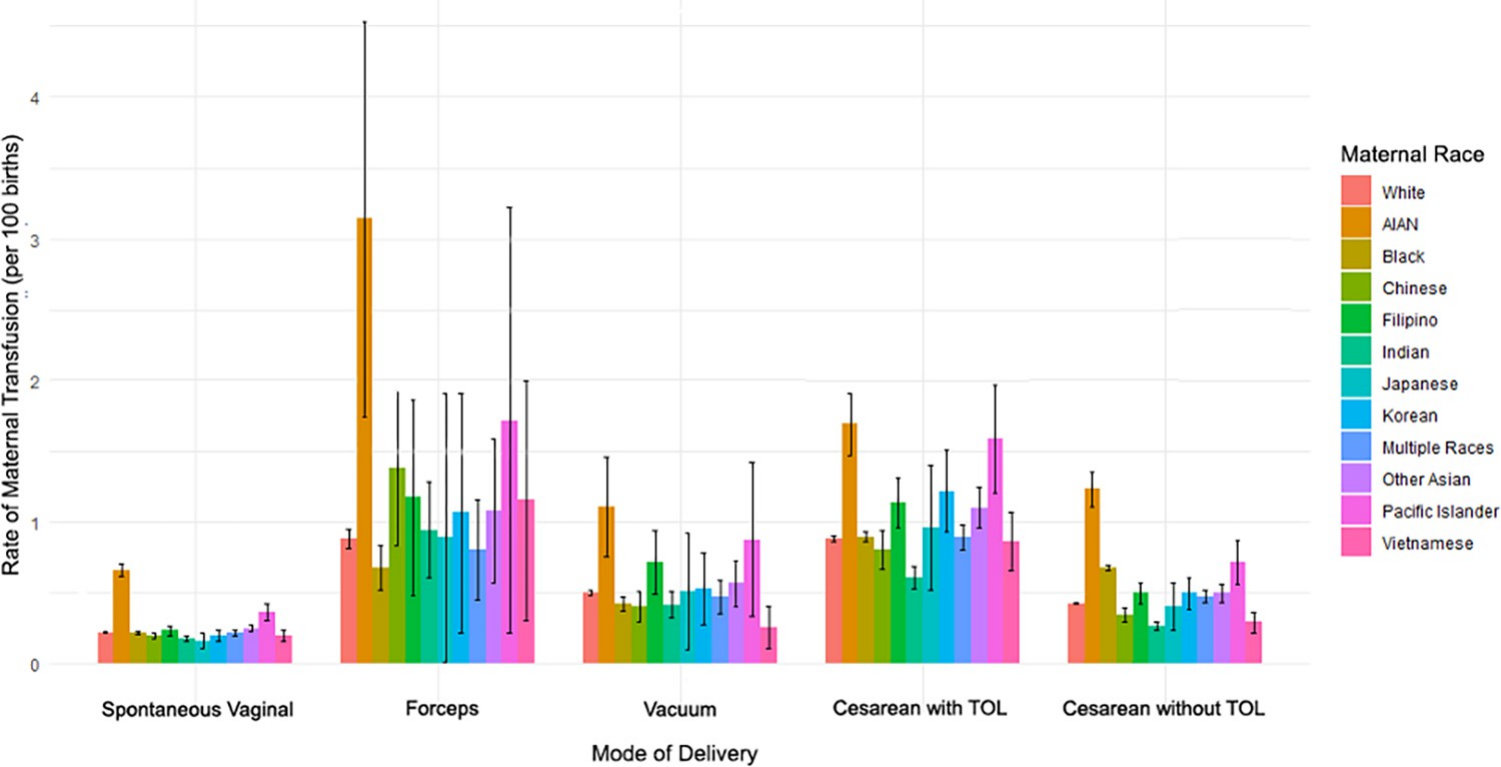
Rate and 95% confidence intervals of maternal blood transfusion by mode of delivery and maternal race.

In the overall population, including all modes of delivery, the rate of maternal blood transfusion was 3.4 per 1,000 deliveries (S1 Table). Compared with the rate of transfusion among White individuals (3.3 per 1,000 deliveries), crude rates of maternal transfusion were higher in AIAN (8.7 per 1,000 births), Black (4.0 per 1,000 births), Filipino (4.1 per 1,000 births), Korean (3.6 per 1,000 births), and Pacific Islander (5.6 per 1,000 births) individuals, as well as in individuals identifying with more than one race (3.9 per 1,000 births) and in individuals identifying as other Asian (3.9 per 1,000 births). Crude rates were lower in Chinese (2.8 per 1,000 births), Indian (2.7 per 1,000 births), Japanese (2.7 per 1,000 births), and Vietnamese (2.8 per 1,000 births) individuals compared to White individuals. The rates of transfusion varied substantially by mode of delivery with the lowest rates found in SVD (2.2 per 1,000 deliveries) and the highest in forceps deliveries (9.0 per 1,000 deliveries; Fig 2 and S1 Table).

### Adjusted association between maternal race and maternal blood transfusion

**Fig 3:**
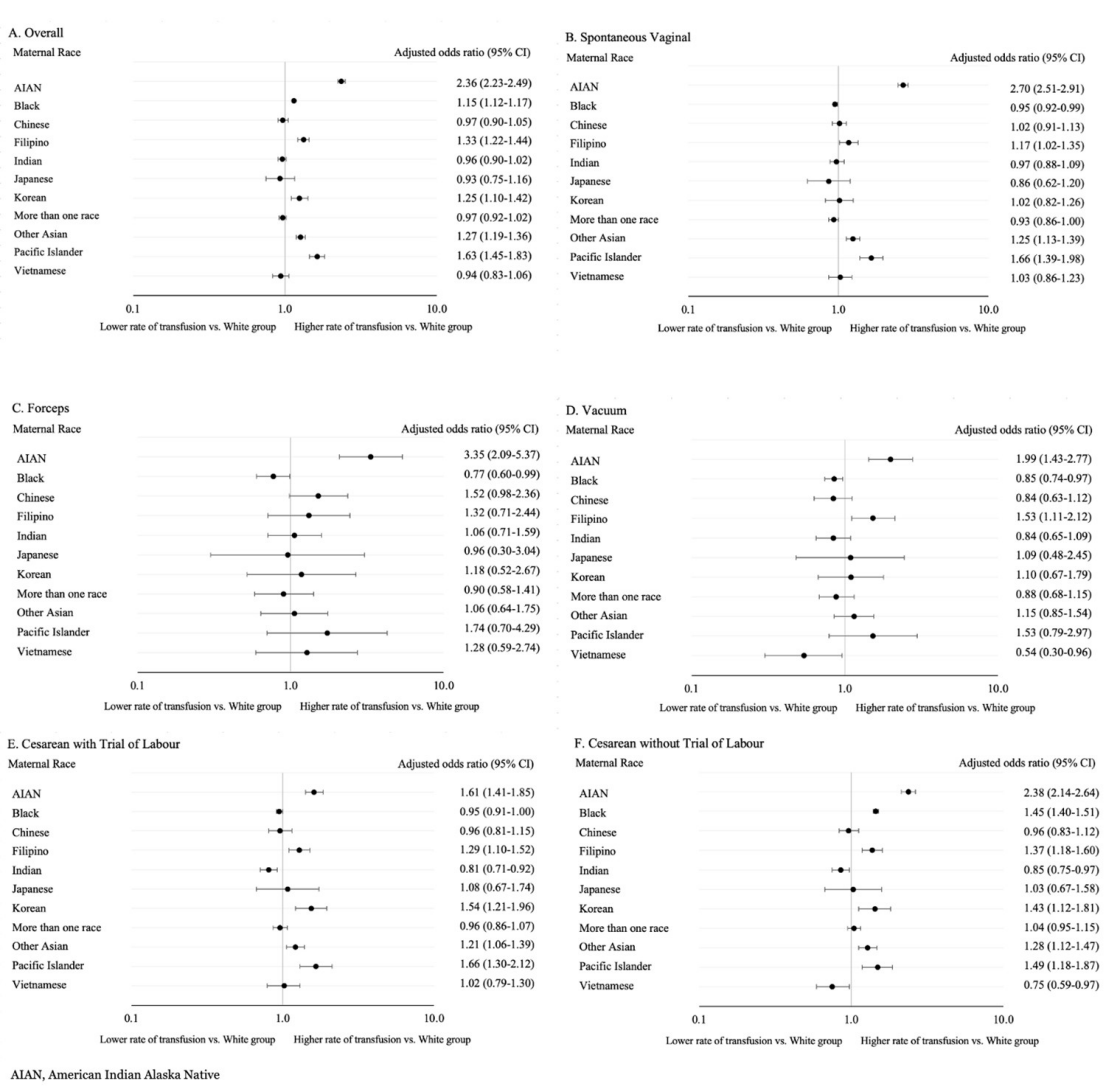
Adjusted odds ratios and 95% confidence intervals expressing the association between maternal race and maternal blood transfusion in the overall population (A), and by mode of delivery (B-F).

In the overall population, higher adjusted odds of transfusion were seen among AIAN (aOR 2.36, 95% CI 2.23–2.49), Pacific Islander (aOR 1.63, 95% CI 1.45–1.83), Filipino (aOR 1.33, 95% CI 1.22–1.44), Korean (aOR 1.25, 95% CI 1.10–1.42), other Asian (aOR 1.27, 95% CI 1.19–1.36), and Black patients (aOR 1.15, 95% CI 1.12–1.17) compared with White patients (Fig 3A).

In the SVD group, the transfusion rate in White patients was 2.2 per 1,000 births. Compared to White patients, the odds of receiving maternal blood transfusion were higher for AIAN (aOR 2.70, 95% CI 2.51–2.91), Filipino (aOR 1.17, 95% CI 1.02–1.35), other Asian (aOR 1.25, 95% CI 1.13–1.39), and Pacific Islander (aOR 1.66, 95% CI 1.39–1.98) individuals (Fig 3B). In contrast, the odds of transfusion were lower for Black patients (aOR 0.95, 95% CI 0.92–0.99) compared with White patients (Fig 3B).

Fewer differences were seen amongst forceps deliveries, where only AIAN (aOR 3.35, 95% CI 2.09–5.37) and Chinese patients (aOR 1.52, 95% CI 1.00–2.36) had greater odds of transfusion compared with White patients. Black patients again had lower odds of transfusion (aOR 0.77, 95% CI 0.60–0.99) compared with White patients (Fig 3C).

With vacuum delivery, AIAN (aOR 1.99, 95% CI 1.43–2.77) and Filipino (aOR 1.53, 95% CI 1.11–2.12) patients experienced higher odds of transfusion compared with White patients while odds were lower for Black (aOR 0.85, 95% CI 0.74–0.97) and Vietnamese patients (aOR 0.54, 95% CI 0.30–0.96; Fig 3D).

The rate of maternal blood transfusion in White patients with a cesarean delivery and TOL was 8.8 per 1,000 births. AIAN (aOR 1.61, 95% CI 1.41–1.85), Filipino (aOR 1.29, 95% CI 1.10–1.52), Korean (aOR 1.54, 95% CI 1.21–1.96), other Asian (aOR 1.21, 95% CI 1.06–1.39) and Pacific Islander (aOR 1.66, 95% CI 1.30–2.12) patients had higher odds of receiving a maternal blood transfusion compared with White patients. Conversely, Black (aOR 0.95, 95% CI: 0.91–1.00) and Indian (aOR 0.81, 95% CI 0.71–0.92) patients were shown to have lower odds of receiving a transfusion compared to White patients in this mode (Fig 3E).

Finally, in the cesarean delivery without TOL analysis, AIAN (aOR 2.38, 95% 2.14–2.64), Black (aOR 1.45, 95% CI 1.4–1.51), Filipino (aOR 1.37, 95% 1.18–1.60), Korean (aOR 1.43, 95% 1.12–1.47), other Asian (aOR 1.28, 95% CI 1.12–1.47), and Pacific Islander (aOR 1.49, 95% CI 1.18–1.87) patients were all shown to experience higher odds of receiving a maternal blood transfusion compared to White patients. Indian (aOR 0.85, 95% CI 0.75–0.97) and Vietnamese (aOR 0.75, 95% CI 0.59–0.97) patients were the only groups shown to have lower odds of transfusion compared to White patients in cesarean deliveries without TOL (Fig 3F).

### Sensitivity analyses

Restricting the study sample to nulliparous individuals resulted in a population of 6,709,378 births and yielded similar results to the main models (S2 Table). The overall rate of maternal transfusion in the nulliparous population was 3.2 per 1,000 with the highest rates experienced by AIAN individuals. The primary difference between these models and the main analysis was the loss of statistical significance in many results due to a widening of confidence intervals (S2 Table).

**Fig 4:**
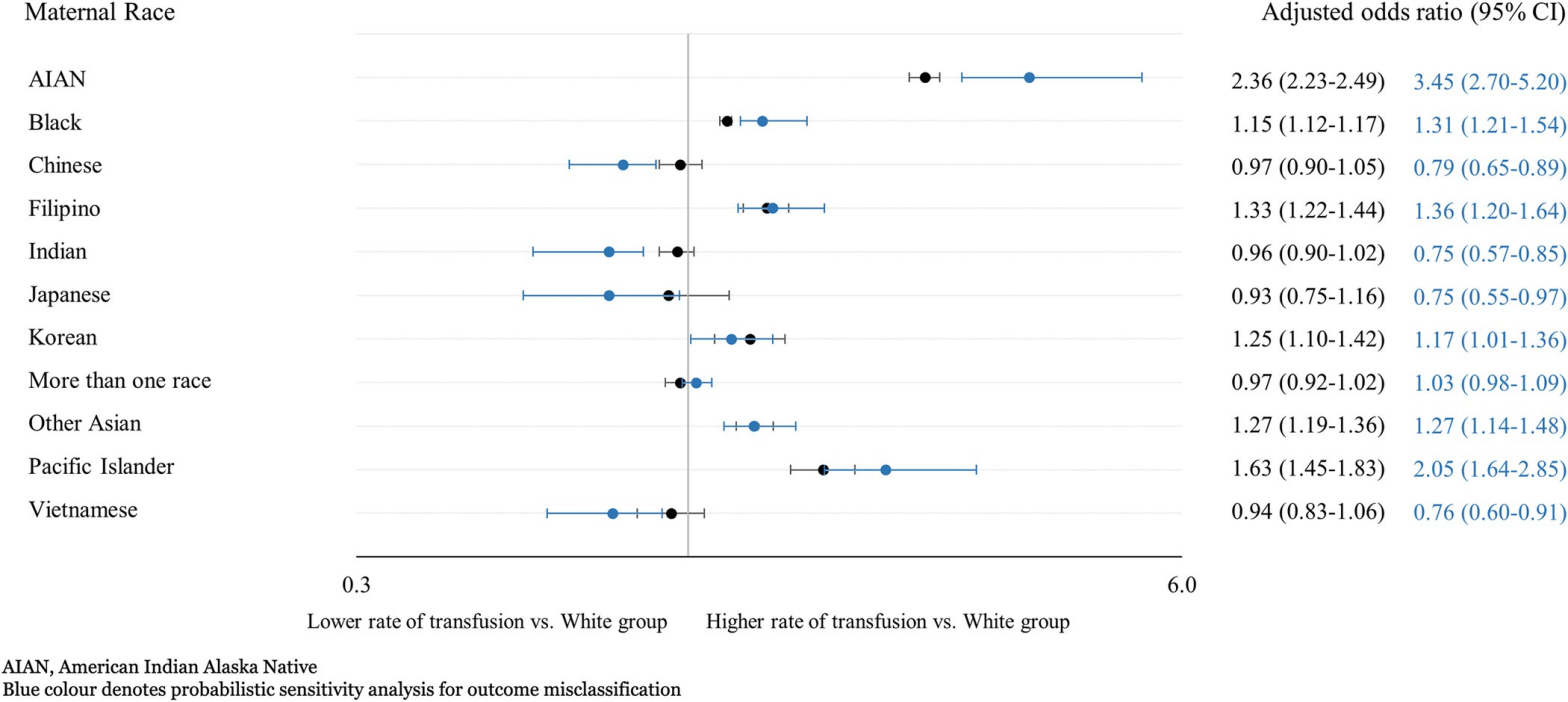
Probabilistic sensitivity analysis correcting for outcome misclassification of maternal blood transfusion by race and mode of delivery.

The results of the probabilistic sensitivity analysis were generally consistent with the main results. While there was a decrease in the rate of transfusion in some groups, this did not change the overall interpretation of our models (Fig 4).

## Discussion

The overall incidence of maternal transfusion in this study population was 3.4 per 1000 births, aligning with published rates [7]. The association between mode of delivery and maternal transfusion documented in the existing literature was affirmed in this cohort, where patients with emergency cesarean deliveries and forceps deliveries received the highest rates of transfusion [10, 11].

Two key findings of this work were the markedly high rates of maternal blood transfusion in AIAN and Pacific Islander patients. AIAN individuals were consistently found to have the highest rates of maternal blood transfusion compared with all other groups regardless of mode of delivery. Pacific Islander patients had similarly high rates, though not as pronounced as those seen with AIAN patients. These findings have been corroborated in the literature, where Pacific Islander patients have been found to have 30–40% increased odds of postpartum hemorrhage compared to White patients [12, 23]. It is important to note, however, that this study did not capture appropriateness of transfusion; nor can this study comment on whether one population received better or worse care from over or under-transfusion. The hemoglobin level and/or presence of symptoms of anemia and/or presence of evidence of end-organ hypoxia were not available in the data source and the findings from our study cannot explain why AIAN or Pacific Islander individuals received more blood transfusions than other racial groups. Rates of antenatal anemia, prevalence of hemoglobinopathy or underlying bleeding disorders, cultural values around blood transfusion, obstetric care differences and resource availability/access are potential factors that could impact blood transfusion rates in a given population. Thus, an important avenue for future research would be to understand the reasons behind the disparate transfusion rates identified in our study.

A recurring theme in the results was the heterogeneity in the rates of transfusion within the Asian racial groups, which are commonly aggregated in the literature as either ‘Asian’ or ‘Asian/Pacific Islander’ [12, 14, 24, 25]. For example, while Vietnamese and Indian individuals consistently had similar or lower adjusted odds of receiving a maternal blood transfusion compared to White individuals, Filipino and Korean patients had consistently higher odds throughout the modes of delivery. These distinctive patterns are overlooked when these groups are combined, resulting in the loss of valuable information that could explain part of what is driving the increasing rates of postpartum hemorrhage in the United States and throughout the world [5].

Another notable finding of this study was that though Black patients had a modest 15% increase in adjusted odds of transfusion compared to White patients in the overall model, stratification by mode of delivery revealed nuanced results. In each delivery mode except cesarean without TOL, Black patients had lower adjusted odds of transfusion compared to White patients. In cesarean deliveries without TOL, this pattern was reversed, and Black patients had a 45% increase in odds of transfusion compared to White patients. Though a prior study set in the United States corroborates the higher rates of postpartum hemorrhage and blood transfusion for Black patients in the overall population [14], we were unable to identify any published work that stratified by mode of delivery when analyzing this relationship. A potential explanation for this finding is that Black patients are more likely to have a cesarean delivery without a trial of labour due to an indication that also increases risk of maternal blood transfusion (e.g., a hemoglobinopathy) [26]. As a result, the rates of blood transfusion in other delivery modes may appear lower because the highest-risk individuals have been excluded before attempting vaginal delivery.

Limitations of this study stem from its retrospective nature and the unavailability of key variables that could help to contextualize our findings. One example is the lack of geographical data prevents us from understanding how rural settings might contribute to the observed disparities. Unavailable data on anemia, blood disorder diagnoses, and details of the transfusions also hinder our ability to interpret the results. While patient income could help mitigate economic influences and allow for a closer examination of racial disparities, we attempted to compensate by incorporating maternal education and WIC receipt into our models. Additionally, there is potential for misclassification of the maternal race in the instances where it was unavailable and paternal race was assigned to the mother.

Strengths of this study include the large patient population facilitating detailed race groupings despite the rarity of the outcome, consistent data collection over study years, the probabilistic sensitivity analysis assessing outcome misclassification, and an abundance of information on many maternal and obstetric characteristics.

## Conclusion

Racial disparities in maternal blood transfusion persist after adjustment for several confounders, particularly within AIAN and Pacific Islander individuals, and vary by mode of delivery. Further research with granular race categories dedicated to understanding the underlying causes behind these trends would be insightful such that these issues be addressed, and transfusion use be prevented and managed in accordance with evidence.

## Supporting information

S1 Table Crude rates of maternal blood transfusion within each mode of delivery by maternal race. (DOCX)

S2 Table Adjusted odds ratios and 95% confidence intervals of maternal blood transfusion compared to White race in nulliparous population. (DOCX)
