# Supplementary material for: Racial disparities in maternal blood transfusion in the United States by mode of delivery
Source: PLoS One. 2024 Oct 21;19(10):e0312110. doi: 10.1371/journal.pone.0312110 (PMC11493266; doi:10.1371/journal.pone.0312110)
Supplement: S1 Table — (DOCX) [file pone.0312110.s001.docx]

Supplementary Materials - Racial disparities in maternal blood transfusion in the United States by mode of delivery

|  | **Overall (N = 17,905,699)** | | | **Spontaneous (n = 11,973,049)** | | | **Forceps (n = 95,410)** | | | **Vacuum (n = 479,546)** | | | **Cesarean with TOL (n = 1,516,827)** | | | **Cesarean without TOL**  **(n = 3,840,867)** | | |
| --- | --- | --- | --- | --- | --- | --- | --- | --- | --- | --- | --- | --- | --- | --- | --- | --- | --- | --- |
| **Race** | **n** | **Rate per 1000** | **95% CI** | **n** | **Rate per 1000** | **95% CI** | **n** | **Rate per 1000** | **95% CI** | **n** | **Rate per 1000** | **95% CI** | **n** | **Rate per 1000** | **95% CI** | **n** | **Rate per 1000** | **95% CI** |
| **Overall (N = 17,905,699)** | 61424 | 3.43 | (3.42,3.43) | 26627 | 2.22 | (2.20, 2.25) | 855 | 8.96 | (8.36, 9.56) | 2352 | 4.90 | (4.71, 5.10) | 13510 | 8.91 | (8.76, 9.06) | 18080 | 4.71 | (4.64, 4.78) |
| **White (n = 13,343,790)** | 43671 | 3.27 | (3.24, 3.30) | 19799 | 2.19 | (2.16, 2.22) | 639 | 8.85 | (8.17, 9.54) | 1735 | 5.01 | (4.77, 5.24) | 9470 | 8.81 | (8.64, 8.99) | 12028 | 4.26 | (4.19, 4.34) |
| **AIAN (n = 162,489)** | 1410 | 8.68 | (8.23, 9.13) | 758 | 6.60 | (6.13, 7.07) | 19 | 31.40 | (17.51, 45.31) | 37 | 11.10 | (7.51, 14.6) | 216 | 16.90 | (14.7, 19.10) | 380 | 12.30 | (11.10, 13.52) |
| **Black (n = 2,669,131)** | 10640 | 3.99 | (3.92, 4.06) | 3675 | 2.16 | (2.09, 2.23) | 74 | 6.78 | (5.24, 8.32) | 257 | 4.23 | (3.71, 4.74) | 2461 | 8.94 | (8.59, 9.29) | 4173 | 6.75 | (6.55, 6.96) |
| **Chinese (n = 269,920)** | 761 | 2.82 | (2.62, 3.02) | 351 | 1.94 | (1.73, 2.14) | 24 | 13.80 | (8.30, 19.30) | 54 | 4.03 | (2.96, 5.11) | 135 | 8.06 | (6.70, 9.41) | 197 | 3.47 | (2.99, 3.96) |
| **Filipino (n = 145,049)** | 589 | 4.06 | (3.73, 4.39) | 209 | 2.32 | (2.01, 2.64) | 11 | 11.70 | (4.82, 18.61) | 40 | 7.15 | (4.94, 9.36) | 156 | 11.30 | (9.57, 13.1) | 173 | 4.97 | (4.23, 5.71) |
| **Indian (n = 362,616)** | 981 | 2.71 | (2.54, 2.87) | 370 | 1.80 | (1.62, 1.99) | 30 | 9.43 | (6.07, 12.82) | 77 | 4.15 | (3.22, 5.07) | 257 | 6.07 | (5.33, 6.81) | 247 | 2.64 | (2.31, 2.97) |
| **Japanese (n = 30,756)** | 84 | 2.73 | (2.15, 3.31) | 35 | 1.60 | (1.07, 2.13) | 3 | 8.96 | (0.00, 19.12) | 6 | 5.09 | (1.02, 9.16) | 18 | 9.56 | (5.16, 14.02) | 22 | 4.03 | (2.35, 5.71) |
| **Korean (n = 69,052)** | 250 | 3.62 | (3.17, 4.07) | 88 | 1.94 | (1.53, 2.34) | 6 | 10.60 | (2.15, 19.11) | 17 | 5.30 | (2.79, 7.81) | 68 | 12.20 | (9.29, 15.00) | 71 | 4.96 | (3.81, 6.11) |
| **More than one race (n = 474,647)** | 1592 | 3.35 | (3.19, 3.52) | 707 | 2.17 | (2.01, 2.33) | 20 | 8.02 | (4.52, 11.50) | 59 | 4.72 | (3.52, 5.92) | 371 | 8.89 | (7.99, 9.79) | 435 | 4.75 | (4.31, 5.20) |
| **Other Asian (n = 229,487)** | 890 | 3.88 | (3.62, 4.13) | 384 | 2.47 | (2.22, 2.72) | 17 | 10.80 | (5.68, 15.90) | 48 | 5.67 | (4.07, 7.27) | 225 | 11.00 | (9.57, 12.41) | 216 | 4.96 | (4.30, 5.62) |
| **Pacific Islander (n = 50,944)** | 284 | 5.57 | (4.93, 6.22) | 126 | 3.63 | (3.00, 4.27) | 5 | 17.20 | (2.16, 32.21) | 10 | 8.76 | (3.34, 14.2) | 67 | 15.90 | (12.1, 19.60) | 76 | 7.16 | (5.56, 8.77) |
| **Vietnamese (n = 97,818)** | 272 | 2.78 | (2.45, 3.11) | 125 | 1.97 | (1.62, 2.31) | 7 | 11.50 | (3.01, 20.00) | 12 | 2.55 | (1.11, 3.98) | 66 | 8.62 | (6.55, 10.73) | 62 | 2.92 | (2.19, 3.64) |

Table S1. Crude rates of maternal blood transfusion within each mode of delivery by maternal race.

AIAN, American Indian and Alaska Native; TOL, trial of labour.
