# Supplementary material for: Racial disparities in maternal blood transfusion in the United States by mode of delivery
Source: PLoS One. 2024 Oct 21;19(10):e0312110. doi: 10.1371/journal.pone.0312110 (PMC11493266; doi:10.1371/journal.pone.0312110)
Supplement: S2 Table — (DOCX) [file pone.0312110.s002.docx]

Supplementary Materials - Racial disparities in maternal blood transfusion in the United States by mode of delivery

Table S2. Adjusted odds ratios and 95% confidence intervals of maternal blood transfusion compared to White race in nulliparous population.

|  | **Overall** | | **Spontaneous** | | **Forceps** | | **Vacuum** | | **Cesarean with TOL** | | **Cesarean without TOL** | |
| --- | --- | --- | --- | --- | --- | --- | --- | --- | --- | --- | --- | --- |
| **Race** | **aOR** | **95% CI** | **aOR** | **95% CI** | **aOR** | **95% CI** | **aOR** | **95% CI** | **aOR** | **95% CI** | **aOR** | **95% CI** |
| **White** | Reference | | Reference | | Reference | | Reference | | Reference | | Reference | |
| **AIAN** | 2.04 | (1.86-2.24) | 2.28 | (2.02-2.59) | 3.32 | (1.91-5.77) | 1.89 | (1.26-2.82) | 1.57 | (1.30-1.90) | 2.17 | (1.59-2.95) |
| **Black** | 0.96 | (0.92-0.99) | 0.84 | (0.80-0.89) | 0.81 | (0.06-1.09) | 0.85 | (0.72-1.00) | 0.98 | (0.92-1.04) | 1.17 | (1.07-1.29) |
| **Chinese** | 0.95 | (0.86-1.05) | 1.06 | (0.92-1.22) | 1.75 | (1.1-2.78) | 0.80 | (0.58-1.12) | 0.96 | (0.78-1.18) | 0.75 | (0.57-0.98) |
| **Filipino** | 1.31 | (1.17-1.48) | 1.16 | (0.95-1.41) | 1.12 | (0.52-2.41) | 1.78 | (1.24-2.55) | 1.36 | (1.12-1.66) | 1.14 | (0.84-1.55) |
| **Indian** | 0.94 | (0.85-1.02) | 0.99 | (0.86-1.14) | 0.94 | (0.58-1.51) | 0.87 | (0.65-1.16) | 0.77 | (0.66-0.91) | 0.78 | (0.61-0.99) |
| **Japanese** | 0.97 | (0.73-1.29) | 0.80 | (0.50-1.27) | 1.29 | (0.40-4.09) | 1.19 | (0.49-2.91) | 1.08 | (0.61-1.92) | 1.23 | (0.68-2.24) |
| **Korean** | 1.22 | (1.04-1.44) | 0.92 | (0.69-1.23) | 1.42 | (0.62-3.26) | 1.11 | (0.65-1.90) | 1.54 | (1.17-2.03) | 1.39 | (0.95-2.01) |
| **More than one race** | 0.90 | (0.84-0.97) | 0.86 | (0.77-0.96) | 0.77 | (0.44-1.34) | 0.93 | (0.69-1.25) | 0.98 | (0.86-1.12) | 0.91 | (0.73-1.14) |
| **Other Asian** | 1.08 | (0.97-1.21) | 1.15 | (0.98-1.35) | 0.59 | (0.28-1.28) | 1.00 | (0.69-1.46) | 1.00 | (0.83-1.21) | 0.94 | (0.68-1.29) |
| **Pacific Islander** | 1.52 | (1.24-1.87) | 1.66 | (1.25-2.21) | 1.14 | (0.28-4.69) | 1.39 | (0.57-3.38) | 1.63 | (1.16-2.31) | 0.68 | (0.28-1.64) |
| **Vietnamese** | 0.83 | (0.69-0.99) | 1.00 | (0.78-1.29) | 1.34 | (0.58-3.07) | 0.29 | (0.12-0.71) | 0.85 | (0.62-1.18) | 0.48 | (0.27-0.85) |

AIAN, American Indian and Alaska Native; TOL, trial of labour.
